# Friedman Score in Relation to Compliance and Treatment Response in Nonsevere Obstructive Sleep Apnea

**DOI:** 10.1155/2020/6459276

**Published:** 2020-03-19

**Authors:** Lars M. Berg, Torun K. S. Ankjell, Yi-Qian Sun, Tordis A. Trovik, Anders Sjögren, Oddveig G. Rikardsen, Ketil Moen, Sølve Hellem, Vegard Bugten

**Affiliations:** ^1^Department of Clinical Dentistry, Faculty of Health Sciences, UiT The Arctic University of Norway, Tromsø, Norway; ^2^ENT Department, University Hospital of Northern Norway, Tromsø, Norway; ^3^Department of Clinical Medicine, Faculty of Health Sciences, UiT The Arctic University of Norway, Tromsø, Norway; ^4^Center for Oral Health Services and Research, Mid-Norway (TkMidt), Trondheim, Norway; ^5^Department of Clinical and Molecular Medicine, Faculty of Medicine and Health Sciences, NTNU Norwegian University of Science and Technology, Trondheim, Norway; ^6^Department of Community Medicine, Faculty of Health Sciences, UiT The Arctic University of Norway, Tromsø, Norway; ^7^ENT Department, Section for Oral and Maxillofacial Surgery, Arendal Hospital, Arendal, Norway; ^8^Department of Clinical Dentistry, Faculty of Medicine, University of Bergen, Bergen, Norway; ^9^Department of Otorhinolaryngology, Head and Neck Surgery, St. Olav's University Hospital, Trondheim, Norway; ^10^Department of Neuromedicine and Movement Science, Faculty of Medicine and Health Sciences, NTNU Norwegian University of Science and Technology, Trondheim, Norway

## Abstract

Nonsevere obstructive sleep apnea (OSA) is most often treated with a continuous positive airway pressure (CPAP) device or a mandibular advancement splint (MAS). However, patient compliance with these treatments is difficult to predict. Improvement in apnea-hypopnea index (AHI) is also somewhat unpredictable in MAS treatment. In this study, we investigated the association between Friedman tongue position score (Friedman score) and both treatment compliance and AHI improvement in patients with nonsevere OSA receiving CPAP or MAS treatment. 104 patients with nonsevere OSA were randomly allocated to CPAP or MAS treatment and followed for 12 months. Data were collected through a medical examination, questionnaires, sleep recordings from ambulatory type 3 polygraphic sleep recording devices, and CPAP recordings. Associations between Friedman score, treatment compliance, and AHI improvement were analysed with logistic regression analyses. Friedman score was not associated with treatment compliance (odds ratio [OR]: 0.85, 95% confidence interval [CI]: 0.59–1.23), or AHI improvement (OR: 1.05, 95% CI: 0.62–1.76) in the overall study sample, the CPAP treatment group, or the MAS treatment group. Adjustment for socioeconomic factors, body mass index, and tonsil size did not significantly impact the results. Although Friedman score may predict OSA severity and contribute to the prediction of success in uvulopalatopharyngoplasty, we found no association between Friedman score and treatment compliance in patients with nonsevere OSA receiving CPAP or MAS treatment, nor did we find any association between Friedman score and AHI improvement. Factors other than Friedman score should be considered when deciding whether a patient with nonsevere OSA should be treated with CPAP or MAS.

## 1. Introduction

Obstructive sleep apnea (OSA) is characterised by breathing cessations during sleep due to transient obstructions in the upper airways [1]. The use of surgical procedures in the upper airways to treat OSA is reserved for only a few, selected patient groups [2, 3]. The most common OSA treatment is continuous positive airway pressure (CPAP). A mandibular advancement splint (MAS) is an alternative for patients with primary snoring or mild OSA or those who are unable or unwilling to use a CPAP device [4, 5]. CPAP treatment significantly lowers the number of breathing cessations in most patients by equalising the negative respiratory pressure that can cause the pharyngeal region to collapse [6]. Unfortunately, poor compliance with CPAP treatment is a significant challenge, especially among patients with nonsevere OSA [7, 8]. Ideally, the CPAP device should be used all night, every night [9]. However, compliance with CPAP treatment is usually regarded as “good” or “adequate” when patients are able to use the CPAP device for more than 4 hours a night [7], at least 70% of nights [10]. MAS treatment, which improves pharyngeal patency by protruding the mandible, shows better compliance, but less predictable improvements in breathing cessations [5, 11–13]. However, both CPAP and MAS treatment can successfully treat nonsevere OSA, as long as patient compliance is adequate [12, 14, 15]. Therefore, tools are needed to help clinicians predict whether the patient is more likely to comply with CPAP or MAS treatment, and if the patient will successfully respond to MAS treatment [16–19]. The Friedman tongue position score (Friedman score) was developed to describe and classify the morphology of the oropharynx with the tongue in a natural relaxed position [20]. A higher Friedman score has been found to predict higher OSA severity [21], which is associated with better compliance with CPAP treatment [8]. In the Friedman Grade staging system, which combines body mass index (BMI), tonsil size, and Friedman score, a low Friedman score has been reported to predict treatment success after uvulopalatopharyngoplasty (UPPP) [2]. In other studies, anatomical obstructions in the nasal cavity and oropharynx have been found to reduce both the effect of and compliance with CPAP treatment [22, 23]. Comparing these findings to the treatment mechanisms of MAS, which relies on relocating the tongue to an anterior position through mandibular protrusion [11], it seems plausible that the Friedman score could be associated with both treatment compliance and apnea-hypopnea index (AHI) improvement in both CPAP and MAS treatment. The Friedman score may therefore be a potential clinical tool for predicting treatment compliance and AHI improvement in CPAP and MAS treatment. In this study, we investigated the association between Friedman score and both treatment compliance and AHI improvement in patients with nonsevere OSA receiving CPAP or MAS treatment.

## 2. Materials and Methods

### 2.1. Study Design and Sample

This prospective, observational study took place in a clinical trial setting and is based on data from a two-centred, parallel-arm randomised controlled trial (RCT), with a 50 : 50 allocation ratio. Due to the nature of CPAP and MAS treatment, the clinical personnel and patients had to know which treatment was received; thus, a blinded RCT was not feasible. All patients in the RCT participated in the current observational study. The patients were recruited to the study after being referred from primary health care to the Ear-Nose-Throat Department of the University Hospital in Northern Norway, Tromsø, and St. Olavs and Aleris Hospitals in Trondheim, Norway. Referred patients were screened for OSA by ambulatory type 3 polygraphic sleep recording devices (Embletta® or Nox T3™, ResMed Norway AS) over night, at home or at a hotel, between October 2014 and February 2018. Resultant sleep recordings were manually analysed by sleep technicians. An otorhinolaryngologist performed a medical examination of the patients and assigned them a Friedman score, which is assessed by a passive, visual inspection of the patient's oral cavity while positioned across from the patient. The 4-grade Friedman score was chosen for this study: grade (I), the entire uvula and palatal tonsils visible; grade (II), the complete soft palate and parts of the uvula visible; grade (III), the uvula not visible and parts of the soft palate visible; and grade (IV), only the hard palate visible [24]. Two researchers (LMB and TKSA) calibrated all involved sleep technicians, dentists, and physicians at the three hospitals according to the study protocol. The protocol checklists complied with updated American Academy of Sleep Medicine practice guidelines for diagnostic testing for OSA [25]. Apnea events were defined as >90% reduction of respiratory flow lasting ≥10 seconds; hypopnea events were defined as ≥50% reduction in respiratory flow lasting ≥10 seconds combined with ≥3% reduction from baseline peripheral blood oxygenation. Nonsevere OSA was defined as AHI <30 events/hour [26].

Inclusion criteria were age 20 to 75 years, AHI between 10.0 and 29.9, and ability to protrude the mandible at least 5 mm. Exclusion criteria were severe OSA (AHI ≥30), pregnancy, drug abuse, daily use of sedative medication, preexisting severe psychiatric disorders, or somatic health issues, such as temporomandibular dysfunction and nasal obstructions, which would interfere with the use of the CPAP device or MAS. Patients who had received previous CPAP or MAS treatment were also excluded.

All patients who met the aforementioned criteria were invited to participate in the study by the otorhinolaryngologist after performing the medical examination. Informed written consent to participate was obtained from 104 patients, who drew lots from a masked envelope for random allocation to either CPAP or MAS treatment. To prevent skewed distribution between treatment groups, across seasons and across study sites, block-randomization with 30 lots per block was used. The number of patients recruited to the study was based on a power calculation for health related quality of life in the RCT which this study was based upon. Baseline characteristics were obtained from a self-administered questionnaire, in which an allergic rhinitis was defined as any respiratory complaint attributed to allergic rhinitis, and smoking was defined as current occasional use or current daily use of smoking tobacco.

The treatment protocol was based on recommendations from the Standards of Practice Committee and the Board of Directors of the American Academy of Sleep Medicine [27]. For patients allocated to the CPAP treatment group, a sleep technician calibrated each CPAP device to the individual patient (Resmed®, San Diego, CA, USA). A facemask or nose mask was used depending on the patient's needs and preference. Patients returned for a follow-up visit 4 months after treatment initiation, during which adjustments were made to the CPAP device if needed, and providers gave patients a motivational talk to advocate the use of the device.

For patients allocated to MAS treatment, a dentist ordered and adapted the MAS (Respire Medical, New York, NY, USA or SomnoDent®, Sydney, NSW, Australia). At treatment initiation, the MAS was set to 60–65% of maximum mandibular protrusion. After 2 to 3 weeks, the MAS was set to the maximum comfortable protrusion, based on feedback from the patient. Patients returned for a follow-up visit 4 months after treatment initiation, during which a new sleep recording was taken while using the MAS, adjustments were made to the MAS if needed, and providers gave patients a motivational talk to advocate its use.

A final follow-up visit occurred at about 12 months after treatment initiation in both treatment groups, at which time all patients completed a questionnaire on treatment compliance. Patients were categorised as compliant if they reported using the CPAP device or MAS more than 4 hours per night, more than 70% of nights [10, 28]. Successful AHI improvement was defined as AHI <10 or AHI <15 when subsequently reducing more than 50% from the AHI at baseline [29].

### 2.2. Statistical Analysis

The associations between Friedman score and treatment compliance and AHI improvement at the final follow-up visit were evaluated with logistic regression in the overall study sample, the CPAP treatment group, and the MAS treatment group. Friedman score was treated as an ordinal variable in the logistic regression analyses, as the associations did not deviate from linearity (*p* > 0.34 for all likelihood ratio tests). The multivariable logistic regression analyses were performed in two models: model 1 was adjusted for sex, age, BMI, education level, and smoking; model 2 was adjusted for all the variables in model 1 as well as tonsil size and was regarded as the main model. The results are presented as odds ratios (ORs) with 95% confidence intervals (CIs) for the outcome per 1-point increase in Friedman score.

All statistical analyses were performed using SPSS 25 statistical software package (IBM Corp, Armonk, NY, USA) and a two-sided *p* < 0.05 was considered statistically significant.

### 2.3. Ethical Approval

The RCT, including the current observational study, was approved by the Norwegian Regional Committee for Medical and Health Research Ethics, REC Central (registration #2014/956) and is registered in ClinicalTrials.gov (registration #NCT02953028).

## 3. Results

**Figure 1:**
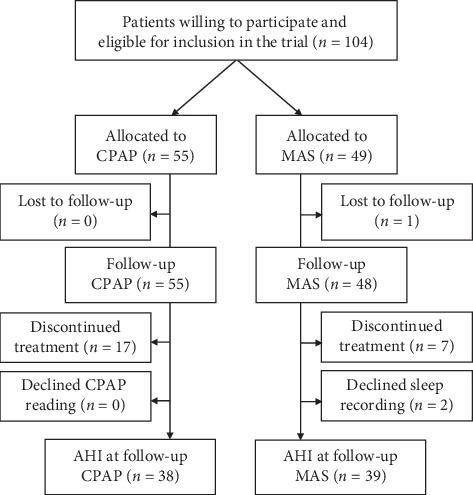
Patient flow chart CPAP: continuous positive airway pressure; MAS: mandibular advancement splint; AHI: apnea-hypopnea index.

Friedman score and baseline characteristics were available for all 104 RCT participants. The final follow-up visit occurred between 10 and 20 months (median 12 month). One patient in the MAS treatment group was lost to follow-up, making compliance data available for 55 and 48 patients in the CPAP and MAS treatment groups, respectively. Prior to follow-up, 24 patients had discontinued treatment and were noncompliant, 17 in the CPAP treatment group and seven in the MAS treatment group. Another two patients in the MAS treatment group declined the sleep recording at final follow-up, despite reporting adequate treatment compliance. Therefore, AHI at final follow-up was available for 38 and 39 patients in the CPAP and MAS treatment groups, respectively (Figure 1).

Baseline patient characteristics were evenly distributed across Friedman scores, except for smoking and tonsil size. Fewer patients with a Friedman score of III were smokers, and more patients with a Friedman score of II had tonsil size grade >1 when compared to those with other Friedman scores (Table 1).

In the logistic regression analyses, Friedman score was not associated with treatment compliance or AHI improvement (Tables 2 and 3). In the main model, the OR for treatment compliance was 0.85 (95% CI 0.59–1.23) per 1-point increase in Friedman score, while the OR for AHI improvement was 1.05 (95% CI 0.62–1.76). No association between Friedman score and treatment compliance/AHI improvement was found when analyses were stratified by treatment group (Tables 4 and 5). The OR for CPAP and MAS treatment compliance was 0.90 (95% CI 0.53–1.54) and 0.98 (95% CI 0.39–2.48), respectively, per 1-point increase in Friedman score. All patients in the CPAP treatment group had an AHI <10 at follow-up; thus no OR was produced for AHI improvement in the CPAP treatment group. OR for MAS treatment was 1.02 (95% CI 0.53–1.98) per 1-point increase in Friedman score.

## 4. Discussion

In this prospective observational study, we found no association between Friedman score and CPAP or MAS treatment compliance. Good treatment compliance is essential for CPAP and MAS treatment to be effective, but achieving adequate compliance is challenging, especially in CPAP treatment [9, 12, 30]. To limit unnecessary treatment failures and poor compliance, tools are needed to guide clinicians to choose which treatment is best suited for each individual patient [18, 19]. Surgical reduction of airway obstructions, including the tongue base, has been shown to increase CPAP treatment compliance [22]. To our knowledge, no previous studies have investigated the direct association between Friedman score and MAS treatment, but a high Friedman score imply that a larger mandibular protrusion might be necessary for successful MAS treatment [31]. Unfortunately, an increased mandibular protrusion is known to increase side effects, which may decrease MAS treatment compliance [11, 32]. Therefore, an association between Friedman score and CPAP and MAS treatment compliance seems plausible. However, when comparing our findings to previous studies, factors such as the patient's and their bed partner's positive attitude towards OSA treatment, patient's increased use of active coping strategies, larger nasal volume and reduced nasal resistance, increased daytime sleepiness, no smoking, and realistic treatment expectations may be better than Friedman score at predicting treatment compliance [7, 8, 30, 33, 34].

Similarly, we found no association between Friedman score and AHI improvement in the CPAP or MAS treatment groups. Previous studies have shown that when combining Friedman score, tonsil size, and BMI into the modified Friedman staging system for patients with OSA [24], lower Friedman score contributes to better results after UPPP in those with nonsevere OSA [3, 23]. Also, surgical reduction of obstructions in the upper airways—such as tonsillectomy in cases of large palatal tonsils [35], or UPPP in cases of large palatal tonsils, excessive tissue in the soft palate, and tongue base [22]—have shown improved AHI and improved CPAP efficacy, particularly in patients with nonsevere OSA. All patients in the CPAP treatment group with AHI measures at final follow-up had successfully reduced their AHI below 10, regardless of their Friedman score at baseline, while patients in the MAS treatment group showed more variation in residual AHI at final follow-up. However, other studies have suggested that younger age, lower BMI, smaller upper airways, less collapsibility in the upper airways, high hyoid bone position, and non-REM dominated and nonpositional OSA may be more important than Friedman score for predicting AHI improvement in MAS treatment [11, 19, 29]. Nevertheless, there are still uncertainties regarding the significance of predictors in successful MAS treatment [19, 36]. Therefore, Friedman score cannot be used to decide whether CPAP or MAS treatment is the most suitable for individual patients with nonsevere OSA.

In our study, we had information on BMI and tonsil size [37]. However, 98.1% of the patients had tonsil size < grade 3, thus limiting our ability to combine Friedman score and tonsil size into the Friedman staging system for patients with OSA in the analyses. Moreover, it is unlikely that the two patients with large palatal tonsils (1.9%) impacted the association between Friedman score and treatment compliance and AHI improvement in this study, even though tonsil size grades 3 and 4 may contribute to nonsevere OSA [35]. All patients in need of surgical intervention that could impact CPAP or MAS treatment were excluded from the RCT this study was based upon, which may have resulted in the skewed distribution of tonsil size in this study. Patients who were likely to benefit from nasal surgery were excluded from our study for the same reason, although OSA patients in general have a more narrow nose than a healthy population [38].

Treatment compliance was significantly lower, while AHI improvement was significantly better in the CPAP treatment group than the MAS treatment group (chi square test, *p* < 0.001). However, the regression analyses in each treatment group showed the same lack of association between Friedman score and treatment compliance/AHI improvement as in the overall study sample. Thus, possible associations in one treatment group were not concealed by the other treatment group in the analysis of the overall study sample. Moreover, the random allocation to the treatment groups ensured that the choice of treatment was not a confounder in the analyses. AHI <10 and AHI <15 with >50% AHI reduction was chosen as criteria of successful AHI improvement, since AHI <15 is likely to present a low risk of health sequelae compared to severe OSA [15, 39–42] and is regarded an adequate goal in MAS treatment [19, 29]. In total, 27 patients did not have an AHI measure at final follow-up. However, 17 had an AHI measure at 4-month follow-up, which was not included in the main analysis. Using these 4-month follow-up measures to replace the missing AHI measures in these 17 patients at final follow-up did not change the lack of association between Friedman score and AHI improvement (Supplementary [Supplementary-material supplementary-material-1]).

We chose not to divide Friedman score II into IIa and IIb as described by Friedman et al. [43]; but this decision is unlikely to impact our results. Due to the inclusion criteria, the relatively small number of patients, and the fact that the patients in the study were recruited following a referral from primary health care, the results from this study may not be generalised to all patients with nonsevere OSA. However, the patients were similar to the Norwegian general population in terms of the demographic variables listed in Table 1, although they had higher BMI and worse self-reported general health at baseline [44, 45]. The patients in our study are probably representative of nonsevere OSA patients without need of nasal or oropharyngeal surgical corrections referred to Norwegian public and private hospitals.

## 5. Conclusions

Although the Friedman score may predict OSA severity and when combined with tonsil size and BMI can predict success in UPPP, we found no association between Friedman score and CPAP and MAS treatment compliance in patients with nonsevere OSA. Neither did we find any association between Friedman score and AHI improvement. Therefore, factors other than Friedman score alone should be considered when deciding whether a patient with nonsevere OSA should be treated with CPAP or MAS.

## Figures and Tables

**Table 1:** Patient characteristics at baseline (*n* = 104).

Friedman score					
Baseline variables	I *n* = 22	II *n* = 23	III *n* = 32	IV *n* = 27	Total
Age at inclusion^*∗*^	52.6 (10.8)	50.4 (11.6)	52.5 (9.1)	50.7 (8.3)	51.7 (9.8)
BMI at inclusion^*∗*^	32.0 (6.8)	28.5 (4.2)	33.2 (8.0)	31.9 (6.2)	31.5 (6.7)
AHI at inclusion^*∗*^	19.1 (6.4)	17.0 (5.6)	18.6 (5.5)	19.2 (5.3)	18.5 (5.6)
Sex					
Female	6 (27.3)	7 (30.4)	14 (43.8)	10 (37.0)	37 (35.6)
Male	16 (72.7)	16 (69.6)	18 (56.3)	17 (63.0)	67 (64.4)
Marital status					
Cohabitating	16 (72.7)	17 (73.9)	25 (78.1)	23 (85.2)	81 (77.9)
Living alone	8 (27.3)	6 (26.1)	7 (21.9)	4 (14.8)	23 (22.1)
Allergic rhinitis					
Yes	5 (22.7)	2 (8.7)	2 (6.3)	8 (29.6)	17 (16.3)
No	17 (77.3)	21 (91.3)	30 (93.8)	19 (70.4)	87 (83.7)
Self-reported health					
Good-excellent	4 (18.2)	8 (34.8)	10 (31.3)	7 (25.9)	29 (27.9)
Poor-fair	18 (81.8)	15 (65.2)	22 (68.8)	20 (74.1)	75 (72.1)
Education level					
College or university	9 (40.9)	13 (56.5)	17 (53.1)	11 (40.7)	50 (48.1)
Other education	13 (59.1)	10 (43.5)	15 (46.9)	16 (59.3)	54 (51.9)
Alcohol consumption					
≤1 time/week	18 (81.8)	18 (78.3)	25 (78.1)	22 (81.5)	83 (79.8)
>1 time/week	4 (18.2)	5 (21.7)	7 (21.9)	5 (18.5)	21 (20.2)
Smoking status					
Nonsmoking	14 (63.6)	18 (78.3)	30 (93.8)	21 (77.8)	83 (79.8)
Smoking	8 (36.4)	5 (21.7)	2 (6.3)	6 (22.2)	21 (20.2)
Tonsil size					
Tonsils absent	2 (9.1)	0 (0)	3 (9.4)	4 (14.8)	9 (8.7)
Grade 1	18 (81.8)	12 (52.2)	20 (62.5)	16 (59.3)	66 (63.5)
Grade 2	1 (4.5)	10 (43.5)	9 (28.1)	7 (25.9)	27 (26.0)
Grade 3	1 (4.5)	1 (4.3)	0 (0)	0 (0)	2 (1.9)
Grade 4	0 (0)	0 (0.0)	0 (0)	0 (0)	0 (0.0)

BMI: body mass index (kg/m2); AHI: apnea-hypopnea index. Tonsil size according to Brodsky grade. ^*∗*^ Mean (standard deviation), all other variables: *n* (%). Allergic rhinitis = any respiratory complaints attributed to allergic rhinitis. Smoking = current occasional or daily use of smoking tobacco.

**Table 2:** Association between Friedman score and treatment compliance evaluated by logistic regression analysis, *n* = 103.

		Treatment compliance (>4 hours, >70% nights)		
	*n* (%)	Crude OR (95% CI)	Model 1 OR (95% CI)	Model 2
				Or (95% CI)
1-point increase in Friedman score	54 (52.4)	0.83 (0.58–1.19)	0.86 (0.60–1.24)	0.85 (0.59–1.23)

*n* (%): using CPAP/MAS >4 hours, >70% of nights, OR: odds ratio, CI: confidence interval. Model 1: adjusted for age, sex, body mass index at inclusion, education level, and smoking. Model 2: adjusted for tonsil size + model 1.

**Table 3:** Association between Friedman score and AHI improvement evaluated by logistic regression analysis, *n* = 77.

		AHI <10 or AHI <15 and reduced >50% at final follow-up		
	*n* (%)	Crude OR (95% CI)	Model 1 OR (95% CI)	Model 2 OR (95% CI)
1-point increase in Friedman score	59 (76.6)	0.99 (0.61–1.60)	1.05 (0.62–1.76)	1.05 (0.62–1.76)

AHI: apnea-hypopnea index. OR: odds ratio, CI: confidence interval. *N* (%): AHI <10 or 15 and reduced >50%. Model 1: adjusted for age, sex, body mass index at inclusion, education level, and smoking. Model 2: adjusted for tonsil size + model 1.

**Table 4:** Association between Friedman score and treatment compliance evaluated by logistic regression analysis, stratified by treatment group, CPAP *n* = 55, MAS *n* = 48.

		Treatment compliance (>4 hours, >70% nights)		
	*n* (%)	Crude OR (95% CI)	Model 1 OR (95% CI)	Model 2 OR (95% CI)
1-point increase in Friedman score, CPAP	18 (32.7)	0.89 (0.53–1.47)	0.96 (0.57–1.62)	0.90 (0.53–1.54)
1-point increase in Friedman score, MAS	36 (75.5)	0.68 (0.35–1.30)	1.03 (0.42–2.52)	0.98 (0.39–2.48)

CPAP: continuous positive airway pressure; MAS: mandibular advancement splint; OR: odds ratio, CI: confidence interval. *N* (%): using CPAP/MAS >4 hours, >70% of nights. Model 1: adjusted for age, sex, body mass index at inclusion, education level, and smoking. Model 2: adjusted for tonsil size + model 1.

**Table 5:** Association between Friedman score and AHI improvement evaluated by logistic regression analysis, stratified by treatment group, CPAP *n* = 38, MAS *n* = 39.

		AHI <10 or AHI <15 and reduced>50% at final follow-up		
	*n* (%)	Crude OR (95% CI)	Model 1 OR (95% CI)	Model 2 OR (95% CI)
1-point increase in Friedman score, CPAP	38 (100)	N.A.	N.A.	N.A.
1-point increase in Friedman score, MAS	21 (53.8)	1.01 (0.56–1.81)	1.02 (0.53–1.98)	1.02 (0.53–1.98)

CPAP: continuous positive airway pressure; MAS: mandibular advancement splint; AHI: apnea-hypopnea index. OR: odds ratio, CI: confidence interval *n* (%): AHI <10 or 15 and reduced >50%. N.A.: all patients achieved AHI <10 at follow-up. Model 1: adjusted for age, sex, body mass index at inclusion, education level, and smoking. Model 2: adjusted for tonsil size + model 1.

## Data Availability

The datasets generated and/or analysed during the current study are not publicly available due to planned publications based on data included in the present datasets but are available from the corresponding author on reasonable request.

## Supplementary Materials

Supplementary Materials Table S1: association between Friedman score and AHI improvement evaluated by logistic regression analysis, missing replaced, *n* = 94.
