# Supplementary material for: Friedman Score in Relation to Compliance and Treatment Response in Nonsevere Obstructive Sleep Apnea
Source: Int J Otolaryngol. 2020 Mar 19;2020:6459276. doi: 10.1155/2020/6459276 (PMC7106919; doi:10.1155/2020/6459276)
Supplement: Supplementary Materials — Table S1: association between Friedman score and AHI improvement evaluated by logistic regression analysis, missing replaced, n = 94. [file 6459276.f1.docx]

## Supplementary Materials

Logistic regression analyses including patients who quit treatment before the final follow-up visit. Missing final follow-up values for these patients were replaced with 4-month follow-up values. No statistically significant associations between Friedman score and AHI improvement was found.

Table S1 Association between Friedman score and AHI improvement evaluated by logistic regression analysis, missing replaced, n=94

|  |  | AHI <10 or AHI <15 and reduced>50% at final follow-up | | |
| --- | --- | --- | --- | --- |
|  | n (%) | Crude  OR (95% CI) | Model 1  OR (95% CI) | Model 2  OR (95% CI) |
| 1-point increase in  Friedman Score | 72 (76.6) | 0.85 (0.54-1.33) | 0.91 (0.56-1.47) | 0.91 (0.56-1.47) |

AHI: apnea-hypopnea index. OR: odds ratio, CI: confidence interval.
n (%): AHI <10 or 15 and reduced >50%.
Model 1: Adjusted for age, sex, body mass index at inclusion, education level and smoking.
Model 2: Adjusted for tonsil size + Model 1.
